# Efficacy of Vitamin D Supplements in Prevention of Acute Respiratory Infection: A Meta-Analysis for Randomized Controlled Trials

**DOI:** 10.3390/nu14040818

**Published:** 2022-02-15

**Authors:** Hae-Eun Cho, Seung-Kwon Myung, Herim Cho

**Affiliations:** 1 Department of Medicine, College of Medicine, Ewha Womans University, Seoul 07804, Korea; puerile97@naver.com (H.-E.C.); herimcho0725@gmail.com (H.C.); 2 Department of Cancer Biomedical Science, National Cancer Center Graduate School of Cancer Science and Policy, Goyang 10408, Korea; 3 Cancer Epidemiology Branch, Division of Cancer Data Science, National Cancer Center Research Institute, Goyang 10408, Korea; 4 Department of Family Medicine and Center for Cancer Prevention and Detection, National Cancer Center Hospital, Goyang 10408, Korea

**Keywords:** vitamin D supplements, acute respiratory infections, randomized controlled trial, meta-analysis

## Abstract

Background: Previous systematic reviews and meta-analyses of randomized controlled trials (RCTs) have reported inconsistent results regarding the efficacy of vitamin D supplements in the prevention of acute respiratory infections (ARIs). Methods: We investigated these efficacy results by using a meta-analysis of RCTs. We searched PubMed, EMBASE, and the Cochrane Library in June 2021. Results: Out of 390 trials searched from the database, a total of 30 RCTs involving 30,263 participants were included in the final analysis. In the meta-analysis of all the trials, vitamin D supplementation showed no significant effect in the prevention of ARIs (relative risk (RR) 0.96, 95% confidence interval (CI) 0.91–1.01, I^2^ = 59.0%, *n* = 30). In the subgroup meta-analysis, vitamin D supplementation was effective in daily supplementation (RR 0.83, 95% CI, 0.73–0.95, I^2^ = 69.1%, *n* = 15) and short-term supplementation (RR 0.83, 95% CI, 0.71–0.97, I^2^ = 66.8%, *n* = 13). However, such beneficial effects disappeared in the subgroup meta-analysis of high-quality studies (RR 0.89, 95% CI, 0.78–1.02, I^2^ = 67.0%, *n* = 10 assessed by the Jadad scale; RR 0.87, 95% CI, 0.66–1.15, I^2^ = 51.0%, *n* = 4 assessed by the Cochrane’s risk of bias tool). Additionally, publication bias was observed. Conclusions: The current meta-analysis found that vitamin D supplementation has no clinical effect in the prevention of ARIs.

## 1. Introduction

Acute respiratory infection (ARI) is classified into an upper respiratory tract infection (URI) and a lower respiratory tract infection (LRI). URIs include common cold (nasopharyngitis), sinusitis, pharyngitis, laryngitis, and laryngotracheitis [1]. The common cold, as a frequent cause of URIs, is caused by viral infections such as rhinovirus, coronavirus, influenza virus A/B/C, respiratory syncytial virus, parainfluenza virus, and adenovirus [1]. URIs are a common disease, of which adults experience 2–4 episodes a year on average, and children 7–12 episodes [2]. LRIs are mostly caused by viruses such as the influenza virus and respiratory syncytial virus. Moreover, they are caused by bacterial infections such as *S. aureus*, *S. pneumoniae*, and *H. influenza*, tuberculosis infections, fungal infections, and parasite infections [3]. LRIs have been the fifth leading cause of death and responsibility for mortality in adults older than 70 years worldwide since 1990, accounting for up to 94.6 per 1000 global deaths [4].

The U.S. Centers for Disease Control and Prevention (CDC) recommends several useful preventive measures for ARIs such as avoiding close contact with a sick person and practicing hygiene: regular handwashing, covering nose and mouth, or using tissues to contain respiratory droplets or secretions. Vaccination against influenza, pneumococcus, and tuberculosis is also being used for the primary prevention of ARIs. Additionally, although oral zinc, vitamin C supplements, vitamin D supplements, ginseng, and probiotics have been suggested to have a preventive effect on the development of ARIs in some studies, this remains inconclusive [5,6], whereas a meta-analysis of six randomized controlled trials (RCTs) reported that lactoferrin supplements, as one of the key immunomodulatory substances, had efficacy in reducing the risk of RTIs [7]. 

In the meantime, it has been reported that vitamin D, which has an important role in calcium and bone homeostasis, affects the immune system [8]. From the previous laboratory studies, 1,25(OH)2D, which is an active form of vitamin D, is related to innate and adaptive immunity: it enhances the antibacterial responses of innate immune cells and inhibits T cell proliferation and cytokine excretion from helper T cells, and downregulates chronic T cell-mediated reactions [8,9,10]. Additionally, an animal study showed that vitamin D could suppress influenza virus replication and inflammation in a mouse model [11]. On the contrary, vitamin D deficiency, which is also associated with nonalcoholic fatty liver disease, obesity, or metabolic syndrome, is known to be linked to an increased risk of infections [12].

Furthermore, several RCTs have reported the preventive effects of vitamin D supplements on the incidence of ARIs [13,14,15,16,17,18,19,20,21,22,23], while others have reported no effect [24,25,26,27,28,29,30,31,32,33,34,35,36,37,38,39,40,41,42]. Several meta-analyses reported whether there are beneficial effects [6,43,44,45,46]. However, Pham et al. [44] and Martineau et al.’s [45] meta-analyses investigated the association between 25(OH)D concentration (not supplementation) and the risk of ARIs but lacked the information about actual doses and regimens for vitamin D supplementation.

The current study aimed to investigate whether vitamin D supplementation reduces the risk of ARIs by using a meta-analysis of RCTs. We conducted various subgroup meta-analyses by important factors such as the duration of supplementation, vitamin dosage, and number of study participants.

## 2. Methods and Materials

### 2.1. Data Sources and Search

We searched the Cochrane Library, Embase, and PubMed in order to retrieve articles about the effect of vitamin D supplementation in the prevention of ARIs from inception to June 2021. Common keywords used for searching were as follows: “vitamin D,” for an intervention variable, “respiratory tract infections,” for a disease variable, and “randomized controlled trial” for a study design. 

### 2.2. Data Selection and Quality

We selected RCTs that met all the following criteria: reported the efficacy of vitamin D supplementation in the prevention of ARIs; reported outcome measures with dichotomous variables. We excluded studies targeting participants in pregnancy and prenatal periods. Regarding studies using shared data from the identical population, we selected a more comprehensive study or a study with a longer follow-up period. Two authors (H.-E. Cho and H. Cho) independently evaluated the suitability of an individual study using the above-described selection criteria. Discrepancies between authors with the selection were solved with discussion and consultation with the third author (S.-K. Myung).

### 2.3. Assessment of Risk of Bias

The risk of bias was estimated based on both the Jadad score [47] and the Cochrane risk of bias tool [48] by two authors (H.-E. Cho and H. Cho). Studies were considered as having high quality if they had ≥5 items in the Jadad scale or ≥6 items in the Cochrane risk of bias tool because the mean score for the Jadad scale was 4.5 and the Cochrane risk of bias tool was 5.

### 2.4. Main and Subgroup Meta-Analysis

In the main analysis, we investigated the association between vitamin D supplementation and the incidence of ARIs as a risk. Subgroup analyses were conducted according to various factors as follows: duration of vitamin D supplementation (≤11 weeks and >11 weeks), dosage (daily, weekly, monthly, >2000 IU, and ≤2000 IU), type of disease (URIs and LRIs), number of the study participants (>1000 vs. ≤1000), region of the study (America, Europe, Asia, and Oceania), mean age (≤18 vs. >18), supply source for supplements (pharmaceutical company vs. non-pharmaceutical company), use of placebo, and quality of the study (Jadad score and Cochrane risk of bias).

### 2.5. Statistical Analysis

Values in cells of a 2 × 2 table based on an intention-to-treat analysis were used to calculate a relative risk (RR) with its 95% confidence interval (CI) in an individual study. Then, we calculated a pooled RR with its 95% CI in the random-effects meta-analysis.

To test the heterogeneity across studies, Higgins I^2^, which measures the percentage of total variation across studies [49], was used. I^2^ calculated by a formula as follows

I^2^ = 100% × (Q − df)/Q (1)

where Q is the Cochrane’s heterogeneity statistic and df means the degree of freedom.

The negative predictive values of the I^2^ were set at zero. An I^2^ value ranges from 0% (no observed heterogeneity) to 100% (maximal heterogeneity), and those greater than 50% indicate substantial heterogeneity [50]. In this study, because individual trials were conducted in the different populations, we used a random-effects model meta-analysis.

The publication bias was evaluated by using the Begg’s funnel plot and Egger’s test. If Begg’s funnel plot shows asymmetry or the *p*-value of the Egger’s test is below 0.05, it indicates the existence of publication bias in the study. We used Stata MP version 17.0 software package (StataCorp., College Station, TX, USA) for all the statistical analyses.

## 3. Results

### 3.1. Identification of Relevant Studies

**Figure 1:**
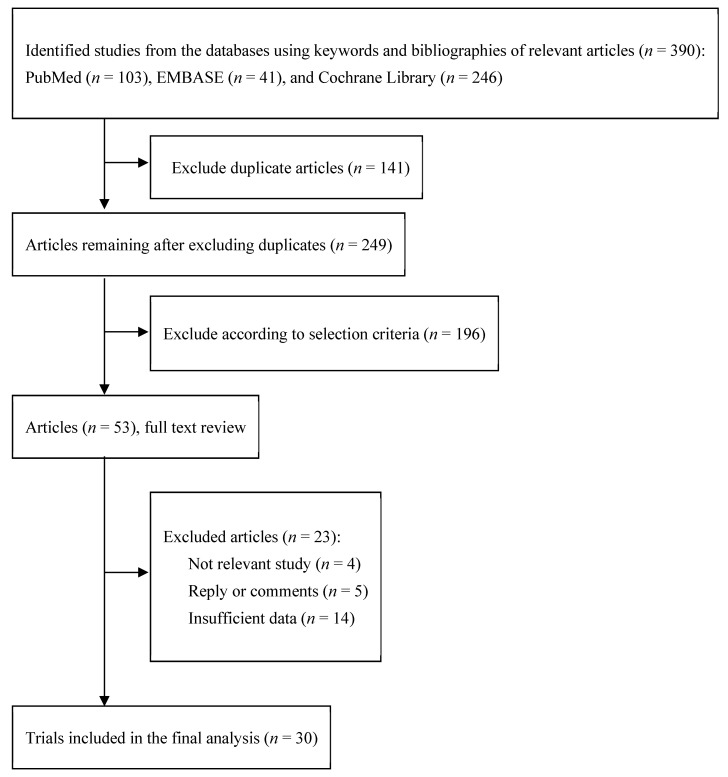
Flow Diagram for selection of relevant clinical trials.

Figure 1 shows how we selected relevant articles, out of a total of 390 articles initially searched from the three databases. After excluding 141 duplicated articles, two authors independently reviewed 249 articles based on the title and abstract. Among them, 196 articles that did not meet the pre-determined selection criteria were excluded. For the remaining 53 articles, we reviewed the full text of the trials and excluded 23 articles because of the following reasons: four articles were irrelevant, five were replies or comments, and 14 had insufficient data. A total of 28 randomized double-blind placebo-controlled trials (RDBPCTs) and two open-label, randomized controlled trials (OLRCTs) [12,13,14,15,16,17,18,19,20,21,22,23,24,25,26,27,28,29,30,31,32,33,34,35,36,37,38,39,40,41] were included in the final analysis.

### 3.2. General Characteristics of Trials

Table 1 shows the general characteristics of the clinical trials included in the final analysis. Studies were published between 2009 and 2021, spanning 12 years. The total number of the study participants were 30,263 with 4259 in an intervention group and 4069 in a control group. The number of the study participants ranged from 49 to 8117. For studies reporting the information of age, the mean age of the participants was 36.6 years old (from 3 to 81). The main outcome measures were URIs (*n* = 23), LRIs (*n* = 6), and both URIs and LRIs (*n* = 1). The periods of supplementation or follow-up ranged from 1 week to 60 weeks.

The dosage regimens for vitamin D supplements used in the trials were as follows: 300, 400, 500, 600, 1000, 1200, 2000, 4000, 10,000 IU daily, 14,000, 50,000 IU weekly, 60,000, 100,000, 120,000, 200,000 IU monthly, 100,000, or 300,000 IU quarterly.

Out of 28 trials reporting their funding sources, eight trials were supplied vitamin D supplements from pharmaceutical companies. The remaining 20 trials were funded by mainly public or governmental organizations or independent scientific foundations.

### 3.3. Association between Vitamin D Supplementation and Prevention of ARIs

**Figure 2:**
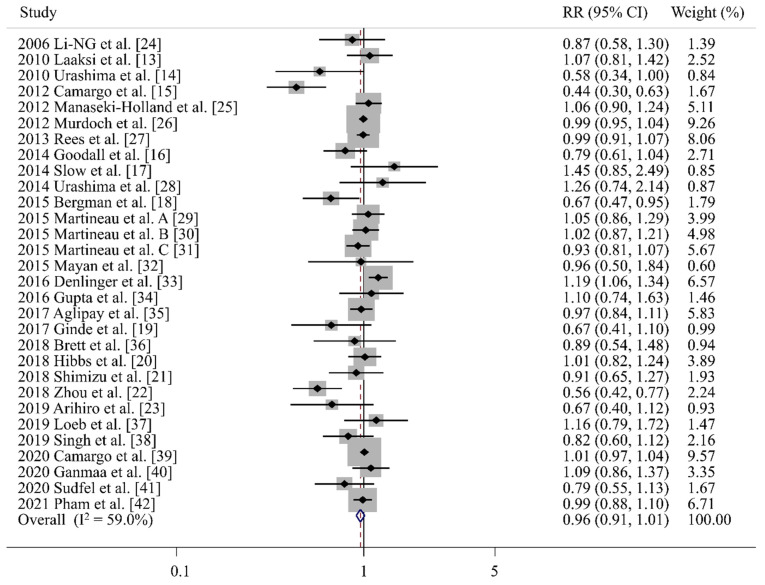
Efficacy of vitamin D supplements in prevention of acute respiratory infections in a meta-analysis of randomized controlled trials (*n* = 30) [13,14,15,16,17,18,19,20,21,22,23,24,25,26,27,28,29,30,31,32,33,34,35,36,37,38,39,40,41,42]. RR, relative risk; CI, confidence interval; A, trial of vitamin D supplementation for prevention of Influenza (ViDiFlu); B, vitamin D3 supplementation in patients with chronic obstructive pulmonary disease (ViDiCO); C, vitamin D3 supplementation in adults with asthma (ViDiAs).

As shown in Figure 2, a random-effects meta-analyses of RCTs showed that vitamin D supplementation did not significantly lower the risk of ARIs (RR 0.96, 95% CI 0.91–1.01, I^2^ = 59.0%, *n* = 30). 

### 3.4. Quality Assessment

The mean score of all the trials was 4.5 (Table 2) and 5 (Table 3) in the quality assessment based on the Jadad scale and the Cochrane risk of bias tool, respectively. Nineteen studies [13,14,15,16,19,20,21,25,26,27,28,30,34,35,37,39,41,42] were considered as having high quality in the Jadad scale, while 11 [17,18,22,23,24,29,32,33,36,38,40] were considered as having low quality (Table 2). Fourteen studies [14,19,23,25,26,28,29,31,34,35,37,39,40,41] were high-quality studies in the Cochrane risk of bias tool, while the remaining 16 [13,15,16,17,18,20,21,22,23,24,27,30,32,33,36,38,42] were low-quality studies (Table 3).

### 3.5. Subgroup Meta-Analysis and the Publication Bias

Table 4 shows that vitamin D supplementation was efficacious in the prevention of ARIs in the subgroup meta-analyses by several factors as follows: duration of the study ≤ 11 weeks, daily supplementation, low vitamin D dosage ≤ 2000 IU, and the number of the study population ≤ 1000.

**Figure 3:**
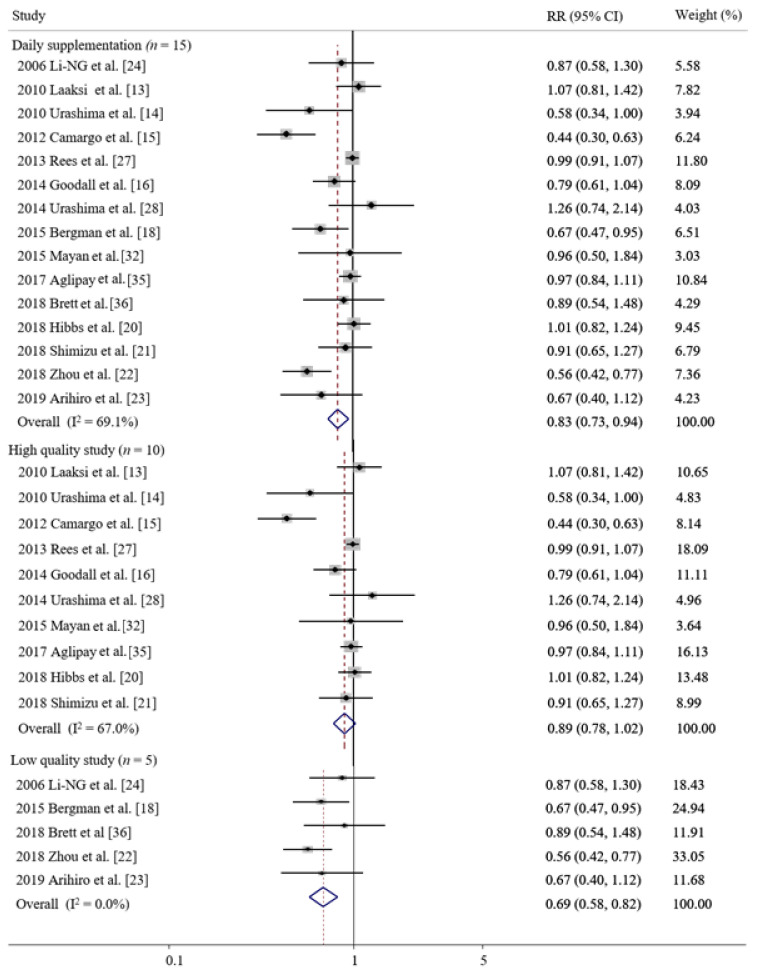
Efficacy of daily supplementation of vitamin D in prevention of acute respiratory infections and its efficacy in subgroup meta-analysis by quality of the study assessed by the Jadad scale [13,14,15,16,18,20,21,22,23,24,27,28,32,35,36]. RR, relative risk; CI, confidence interval.

**Figure 4:**
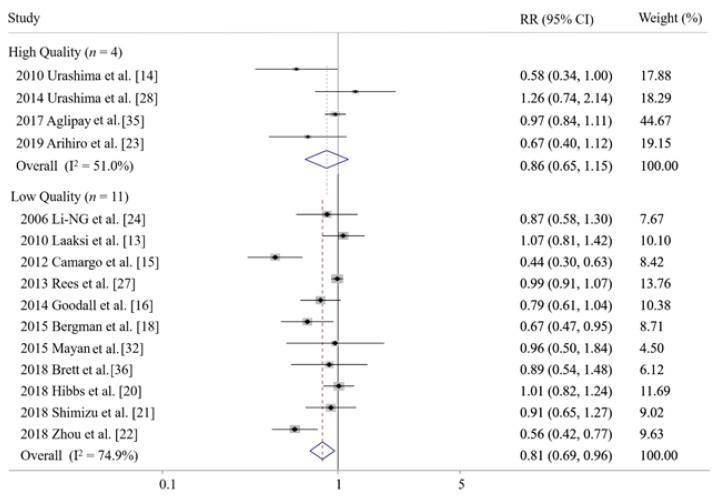
Efficacy of daily supplementation of vitamin D in prevention of acute respiratory infections in subgroup meta-analysis by quality of the study assessed by Cochrane’s risk of bias tool [13,14,15,16,18,20,21,22,23,24,27,28,32,35,36]. RR, relative risk; CI, confidence interval.

Daily supplementation of vitamin D significantly decreased the risk of ARIs (RR 0.83, 95% CI 0.73–0.95, I^2^ = 69.1%, *n* = 15, Figure 3), while its weekly and monthly supplementation showed no significant association. However, in the subgroup meta-analysis of high-quality studies, beneficial effects of daily vitamin D were not observed (RR 0.89, 95% CI, 0.78–1.02, I^2^ = 67.0%, *n* = 10, assessed by the Jadad scale, Figure 3; RR 0.86, 95% CI, 0.65–1.15, I^2^ = 51.0%, *n* = 4, assessed by the Cochrane’s risk of bias tool, Figure 4), while beneficial effects remained in low-quality studies (Figure 3 and Figure 4).

In the subgroup meta-analysis by the duration of vitamin D supplementation, the short-term use of vitamin D supplements showed a significant decreased risk of ARIs in the short-term (RR 0.83, 95% CI, 0.71–0.97, I^2^ = 66.8%, *n* = 13, Table 4). Similar to daily supplementation of vitamin D, beneficial effects disappeared in the subgroup meta-analysis of high-quality studies (RR 0.88, 95% CI, 0.73–1.05, I^2^ = 68.7%, *n* = 9, assessed by the Jadad scale; RR 0.93, 95% CI, 0.74–1.16, I^2^ = 43.6%, *n* = 5, assessed by the Cochrane’s risk of bias tool, Table 4), while beneficial effects remained in low-quality studies (Table 4).

**Figure 5:**
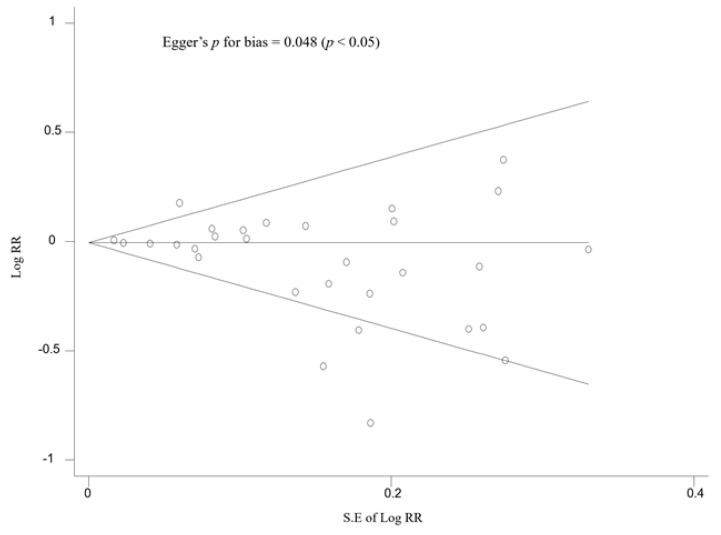
Begg’s funnel plot and Egger’s test for identifying publication bias of randomized controlled trials. RR, relative risk; S.E, standard error.

As shown in Figure 5, publication bias was observed: the Begg’s funnel plot was asymmetrical, and the Egger’s *p* for bias was 0.048 (*p* < 0.05).

## 4. Discussion

In the current study, we found that the use of vitamin D supplements had no preventive effect on ARIs in the meta-analysis of 30 RCTs. Vitamin D supplementation was efficacious in the prevention of ARIs in the subgroup meta-analyses in daily supplementation and its short-term use. However, the subgroup meta-analyses of the high-quality studies in each category showed that the use of vitamin D supplements has no statistically significant effect in the prevention of ARIs.

There are several biological mechanisms that could explain the preventive effect of vitamin D supplements on ARIs. It has been reported that vitamin D modulates both the adaptive immune and innate immune systems from in vitro studies and animal studies. First, vitamin D could work as a direct and indirect regulator of T cells [7]. Vitamin D regulates T cells directly by inhibiting T cell proliferation, Interleukin-2 (IL-2) and Interferon-γ (INF-γ) transcription, and IL-17 secretion by helper T 17 cells. Additionally, the vitamin D receptor (VDR) is expressed in both the innate and the adaptive immune cells [7]. The VDR mediates 1,25(OH)2D to suppress helper T 1 cell proliferation that produces inflammatory cytokines, thus decreasing the production of INF-γ and IL-2 [51,52]. Moreover, vitamin D induces the development of IL-10 and regulatory T cells [8]. Second, vitamin D fortifies the antibacterial responses of the innate immune response by the toll-like receptors (TLRs) and the 1,25(OH)2D/VDR signaling [7]. The TLRs, which are expressed on macrophages, polymorphonuclear cells, monocytes, and epithelial cells play a key role in the innate immune system [50]. Some of the antimicrobial peptides that demonstrate antiviral effects are associated with the TLRs, and their expression is affected by 1,25(OH)2D [7,50]. In addition, several TLRs are affected by the VDR stimulation [50]. Finally, the gene expression of the antibacterial agents, cathelicidin, and human β-defensin are induced by 1,25(OH)2D/VDR signaling [7]. Cathelicidin is an antimicrobial peptide induced by the TLR 1/2 activation, and human β-defensin acts as a chemoattractant for neutrophils and monocytes [50]. In the animal study, the lungs of the 25(OH)D3-fed mice had a significantly lower viral titer than the lungs of the control mice. After influenza virus infection, the proinflammatory cytokines, IL-5 and INF-γ, significantly decreased in 25(OH)D3-fed mice compared with the control mice. 25(OH)D3 was found to reduce viral replication and inflammatory cytokines, and then decreased the clinical manifestation of influenza virus infection in a mouse model [11]. In other words, vitamin D deficiency is associated with an increased risk of infections of bacterial and viral origin through decreased innate immunity [53].

In the meantime, previous RCTs and meta-analyses have reported inconsistent findings about the preventive effect of vitamin D supplements on ARIs [6,43,44,45,46]. Among them, only one study reported consistent findings with ours [42], and the others reported a preventive effect of vitamin D on ARIs [6,44,45,46]. Xiao et al.’s [43] systematic review in 2015 concluded that there was no efficacy of vitamin D supplementation for the prevention of childhood ARIs. Martineau et al. [44] and Pham et al. [45] reported that high levels of serum 25(OH)D are associated with the prevention of ARIs. Abioye et al. [6] reported that micronutrients including vitamin D, vitamin C, and zinc reduced the occurrence of ARIs and the duration of the symptoms. Jolliffe et al. [46] suggested that although the heterogeneity across the trial was significant, the vitamin D supplementation slightly reduced the risk of ARIs compared to the control group.

Compared to the previous meta-analyses, our study has several strengths. We conducted subgroup meta-analyses by important factors that affect individual results and found out that the preventive effect of vitamin D supplements on ARIs was associated with the quality of the studies. In the subgroup meta-analysis, a significant preventive effect of vitamin D supplementation on ARIS was observed in daily supplementation and in the use of supplements during the short-term period. However, such beneficial effects disappeared in the subgroup meta-analysis of high-quality studies. That is, we think that the inconsistent findings of the previous meta-analyses might be associated with the study quality. Moreover, we used both the Jadad scale and Cochrane risk of bias tool to assess the methodological quality of the trials. Because the Jadad scale, which is a simple tool for assessing study quality, has been criticized by its generic problems of scale, we also used the Cochrane risk of bias tool for accuracy.

There are some limitations in this study. First, it would be ideal to investigate the efficacy of vitamin D supplementation on ARIs considering the baseline concentration of the 25(OH)D. However, this was unavailable in most of the studies included in our analysis. Thus, we could not investigate the differences in the preventive effect on ARIs between people with vitamin D deficiency and normal vitamin D levels. Further clinical trials with the data of baseline 25(OH)D levels are warranted to confirm our findings. Second, publication bias was found in this study, which means that trials showing an increasing risk of or no effect on ARIs by vitamin D supplementation might not be published. This favors our conclusion that there is no preventive effect of vitamin D supplements on ARIs. Finally, several RCTs included in the current study were not designed specifically to investigate the efficacy of vitamin D supplements on ARIs as a primary endpoint. Findings in the secondary endpoint might be due to chance.

## 5. Conclusions

The current meta-analysis of RCTs shows that the use of vitamin D supplements has no efficacy in the prevention of ARIs.

## Figures and Tables

**Table 1:** Characteristics of trials included in the final meta-analysis (*n* = 30).

	Study	Region	Study Design (Type of Prevention)	Participants (Average Age, y; Women, %)	Duration of Supplementation, w (Follow-Up Period, w)	Intervention vs. Control	Main Outcome Measures	No. of Patients with Acute Respiratory Infection /No. of Study Participants	
								Supplement Group	Control Group
1	2009, Li-Ng et al. [24]	U.S.	RDBPCT	148 healthy adults (59; 80)	3 (3)	Vitamin D (2000 IU/d) vs. placebo	URI symptoms	28/78	29/70
2	2010, Laaksi et al. [13]	Finland	RDBPCT	164 healthy young men with military training (n.a.; 0)	6 (6)	Vitamin D (400 IU/d) vs. placebo	common cold symptoms	45/80	44/84
3	2010, Urashima et al. [14]	Japan	RDBPCT	334 Children (10; 44)	4 (4)	Vitamin D (1200 IU/d) vs. placebo	Influenza A infection	18/167	31/167
4	2012, Camargo et al. [15]	Mongolia	RDBPCT	244 Children (10; 48)	3 (3)	Vitamin D (300 IU/d) + milk vs. milk	Acute respiratory infection	31/141	52/103
5	2012, Manaseki et al. [25]	Afghanistan	RDBPCT	3046 healthy infants (n.a.; 48)	18 (18)	Vitamin D (100,000 IU/3 m) vs. placebo	pneumonia with CXR	260/1524	245/1522
6	2012, Murdoch et al. [26]	New Zealand	RDBPCT	322 healthy adults (48; 75)	18 (18)	Vitamin D (100,000 IU/m) vs. placebo	URI symptoms	154/161	155/161
7	2013, Rees et al. [27]	n.a.	RDBPCT	759 healthy adults with history of colorectal adenoma (58; 42)	n.a.	Vitamin D (1000 IU/d) vs. placebo	URI symptoms	303/399	276/360
8	2014, Goodall et al. [16]	U.S.	RDBPCT	492 healthy students (19; 64)	1 (1)	Vitamin D (10,000 IU/d) vs. placebo	URI symptoms	70/258	80/234
9	2014, Slow et al. [17]	New Zealand	RDBPCT	207 non-*S. aureus* nasal carriage adults (48; 75)	18 (18)	Vitamin D (100,000 IU/m) vs. placebo	*S. aureus* nasal carriage, culture positive	28/110	17/97
10	2014, Urashima et al. [28]	Japan	RDBPCT	247 adolescents never have Influenza A (n.a.; 34)	2 (2)	Vitamin D (2000 IU/d) vs. placebo	Influenza-like illness	32/148	17/99
11	2015, Bergman et al. [18]	Sweden	RDBPCT	124 patients with primary immunodeficiency (n.a.; n.a.)	12 (12)	Vitamin D (4000 IU/d) vs. placebo	URI symptoms	26/62	39/62
12	2015, Martineau et al. A (ViDiFlu) [29]	U.K.	RDBPCT	217 residents of sheltered accommodation housing blocks (67; 66)	12 (12)	Vitamin D (120,000 IU/2 m + 400 IU/d) vs. Vitamin D ( 400 IU/d) + placebo	ARI symptoms	83/125	58/92
13	2015, Martineau et al. B (ViDiCO) [30]	U.K.	RDBPCT	205 patients with COPD, emphysema, chronic bronchitis (65; 40)	12 (12)	Vitamin D (120,000 IU/2 m) vs. placebo	URI symptoms	76/102	75/103
14	2015, Martineau et al. C (ViDiAs) [31]	U.K.	RDBPCT	232 patients with asthma (48; 57)	12 (12)	Vitamin D (120,000 IU/2 m) vs. placebo	URI symptoms	85/115	93/117
15	2015, Mayan et al. [32]	Israel	RDBPCT	55 adolescent swimmers (15; 36)	12 (12)	Vitamin D (2000 IU/d) vs. placebo	URI symptoms	11/28	11/27
16	2016, Denlinger et al. [33]	n.a.	RDBPCT	408 patients with asthma (n.a.)	28 (28)	Vitamin D (4000 IU/d) vs. placebo	URI symptoms	161/201	139/207
17	2016, Gupta et al. [34]	India	RDBPCT	314 children with pneumonia (12 m; 30)	once (6)	Vitamin D (100,000 IU) vs. placebo	pneumonia	39/156	36/158
18	2017, Aglipay et al. [35]	Canada	RDBPCT	703 healthy children (3; 42)	4–8 (4–8)	Vitamin D (2000 IU/d + 400 IU/d) vs. Vitamin D (400 IU/d)	URI	184/349	193/354
19	2017, Ginde et al. [19]	U.S.	RDBPCT	107 long term care residents (81; 58)	12 (12)	Vitamin D (100,000 IU/m) vs. Vitamin D (1200 IU/m)	URI symptoms	17/55	24/52
20	2018, Brett et al. [36]	Canada	OLRCT	49 healthy children (6; 47)	3 (3)	Vitamin D fortified food (600 IU/d) vs. placebo	common cold symptoms	13/25	14/24
21	2018, Hibbs et al. [20]	U.S.	RDBPCT	306 preterm black infants (n.a.; 67)	6 (12)	Vitamin D (400 IU/d) vs. placebo	URI	84/153	83/153
22	2018, Shimizu et al. [21]	Japan	RDBPCT	215 healthy adults (54; 69)	16 (16)	Vitamin D (400 IU/d) vs. placebo	URI symptoms	41/110	43/105
23	2018, Zhou et al. [22]	China	OLRCT	332 healthy infants (8; 48)	4 (4)	Vitamin D (1200 IU/d + 400 IU/d) vs. Vitamin D (400 IU/d)	Influenza A	43/164	78/168
24	2019, Arihiro et al. [23]	Japan	RDBPCT	223 patients with IBD (45; 39)	6 (6)	Vitamin D (500 IU/d) vs. placebo	URI symptoms	19/108	30/115
25	2019, Loeb et al. [37]	Vietnam	RDBPCT	1300 healthy children and adolescent (9; 52)	8 (8)	Vitamin D (14,000 IU/w) vs. placebo	Influenza A or B	50/650	43/650
26	2019, Singh et al. [38]	n.a.	OLRCT	100 children with pneumonia (n.a.; 42)	8 (12)	Vitamin D (300,000/3 m) + milk vs. placebo + milk	LRI symptoms	28/50	34/50
27	2020, Camargo et al. [39]	New Zealand	RDBPCT	5056 healthy adults (66; 42)	19.2 (19.2)	Vitamin D (100,000 IU/m) vs. placebo	ARI symptoms	1882/2539	1855/2517
28	2020, Ganmaa et al. [40]	Mongolia	RDBPCT	8117 children without TB (9; 49)	36 (36)	Vitamin D (14,000 IU/w) vs. placebo	Pulmonary TB, QFT results	147/4074	134/4043
29	2020, Sudfeld et al. [41]	Tanzania	RDBPCT	3639 patients with HIV with ART (39; 32)	12 (12)	Vitamin D (50,000 IU/w than 2000 IU/d) vs. placebo	Pulmonary TB	50/1812	64/1827
30	2021, Pham et al. [42]	Australia	RDBPCT	2598 healthy adults (n.a.; 51)	60 (60)	Vitamin D (60,000 IU/m) vs. placebo	ARI symptoms	410/1318	404/1280

n.a., not available; RDBPCT, randomized, double-blind, placebo-controlled trial; OLRCT, open-label, randomized, controlled trial; URI, upper respiratory tract infection; LRI, lower respiratory tract infection; ARI; acute respiratory infection; TB, tuberculosis; CXR, chest X-ray; QFT, QuantiFERON-TB; ART, antiretroviral therapy; HIV, human immunodeficiency virus; A (ViDiFlu), trial of vitamin D supplementation for prevention of Influenza; B (ViDiCO), vitamin D3 supplementation in patients with chronic obstructive pulmonary disease; C (ViDiAs), vitamin D3 supplementation in adults with asthma.

**Table 2:** Methodological quality of trials based on the Jadad scale (*n* = 30).

	Study	Randomization	Description of Randomization Methods	DOUBLE-BLIND	Using Identical Placebo	Follow-Up Reporting	Total Score
1	2009, Li-Ng et al. [24]	1	1	1	0	1	4
2	2010, Laaksi et al. [13]	1	1	1	1	1	5
3	2010, Urashima et al. [14]	1	1	1	1	1	5
4	2012, Camargo et al. [15]	1	1	1	1	1	5
5	2012, Manaseki et al. [25]	1	1	1	1	1	5
6	2012, Murdoch et al. [26]	1	1	1	1	1	5
7	2013, Rees et al. [27]	1	1	1	1	1	5
8	2014, Goodall et al. [16]	1	1	1	1	1	5
9	2014, Slow et al. [17]	1	0	1	1	1	4
10	2014, Urashima et al. [28]	1	1	1	1	1	5
11	2015, Bergman et al. [18]	1	1	1	0	1	4
12	2015, Martineau et al. A (ViDiFlu) [29]	1	0	1	0	1	3
13	2014, Martineau et al. B (ViDiCO) [30]	1	1	1	1	1	5
14	2015, Martineau et al. C (ViDiAs) [31]	1	1	1	1	1	5
15	2015, Mayan et al. [32]	1	0	1	1	1	4
16	2016, Denlinger et al. [33]	1	0	1	0	1	3
17	2016, Gupta et al. [34]	1	1	1	1	1	5
18	2017, Aglipay et al. [35]	1	1	1	1	1	5
19	2017, Ginde et al. [19]	1	1	1	1	1	5
20	2018, Brett et al. [36]	1	0	0	0	0	1
21	2018, Hibbs et al. [20]	1	1	1	1	1	5
22	2018, Shimizu et al. [21]	1	0	1	1	1	5
23	2018, Zhou et al. [22]	1	0	0	0	1	2
24	2019, Arihiro et al. [23]	1	1	1	0	1	4
25	2019, Loeb et al. [37]	1	1	1	1	1	5
26	2019, Singh et al. [38]	1	0	0	1	1	3
27	2020, Camargo et al. [39]	1	1	1	1	1	5
28	2020, Ganmaa et al. [40]	1	0	1	0	1	3
29	2020, Sudfeld et al. [41]	1	1	1	1	1	5
30	2021, Pham et al. [42]	1	1	1	1	1	5

**Table 3:** Methodological quality of trials based on the Cochrane risk of bias tool (*n* = 30).

Study	Random Sequence Generation	Allocation Concealment	Blinding of Participants and Personnel	Blinding of Outcome Assessment	Incomplete Outcome Data	Selective Reporting	Other Bias	No. of Low Risk of Bias
2009, Li-Ng et al. [24]	Low	Unclear	Low	High	Low	Low	Low	5
2010, Laaksi et al. [13]	Low	Low	Unclear	Low	Unclear	Low	Low	5
2010, Urashima et al. [14]	Low	Low	Low	Low	Unclear	Low	Low	6
2012, Camargo et al. [15]	Low	Low	Low	Unclear	Low	Unclear	Low	5
2012, Manaseki et al. [25]	Low	Low	Low	Low	Unclear	Low	Low	6
2012, Murdoch et al. [26]	Low	Low	Low	Low	Low	Low	Low	7
2013, Rees et al. [27]	Low	Low	Low	High	Unclear	Low	Low	5
2014, Goodall et al. [16]	Low	Low	Low	Low	Unclear	Unclear	Low	5
2014, Slow et al. [17]	Unclear	Unclear	Low	Low	Low	Low	Low	5
2014, Urashima et al. [28]	Low	Low	Low	Unclear	Low	Low	Low	6
2015, Bergman et al. [18]	Low	Low	Low	Low	Unclear	Unclear	Low	5
2015, Martineau et al. A (ViDiFlu) [29]	Low	Unclear	Low	Low	Low	Low	Low	6
2014, Martineau et al. B (ViDiCO) [30]	Low	Unclear	Low	Unclear	Low	Low	Low	5
2015, Martineau et al. C (ViDiAs) [31]	Low	Low	Low	Unclear	Low	Low	Low	6
2015, Mayan et al. [32]	Unclear	High	Unclear	Unclear	Unclear	Low	Low	2
2016, Denlinger et al. [33]	Unclear	Unclear	Unclear	Unclear	Unclear	Low	Low	2
2016, Gupta et al. [34]	Low	Low	Low	Low	Low	Low	Low	7
2017, Aglipay et al. [35]	Low	Low	Low	Low	Unclear	Low	Low	6
2017, Ginde et al. [19]	Low	Low	Low	Low	Low	Low	Low	7
2018, Brett et al. [36]	Unclear	Unclear	Unclear	Unclear	Unclear	Low	Low	2
2018, Hibbs et al. et al. [20]	Low	Unclear	Low	Unclear	Low	Low	Low	5
2018, Shimizu et al. [21]	Low	Low	Low	Unclear	Unclear	Low	Low	5
2018, Zhou et al. [22]	Unclear	Unclear	Unclear	Unclear	Unclear	Low	Low	2
2019, Arihiro et al. [23]	Low	Low	Low	Low	Low	Low	Low	7
2019, Loeb et al. [37]	Low	Low	Low	Low	Unclear	Low	Low	6
2019, Singh et al. [38]	Unclear	Low	Unclear	Unclear	Unclear	Low	Low	3
2020, Camargo et al. [39]	Low	Low	Low	Unclear	Low	Low	Low	6
2020, Ganmaa et al. [40]	Low	Unclear	Low	Low	Low	Low	Low	6
2020, Sudfeld et al. [41]	Low	Low	Low	Low	Low	Low	Low	7
2021, Pham et al. [42]	Low	Low	Low	Unclear	Unclear	Low	Low	5

**Table 4:** Vitamin D supplementation in prevention of acute respiratory infections in the subgroup meta-analysis of randomized controlled trials by various factors.

Factors	No. of Trials	Summary RR (95% CI)	Heterogeneity, I^2^ (%)
All	30	0.96 (0.91–1.01)	59.0
Duration of Vitamin D supplementation			
Long term	15	1.01 (0.9–1.06)	38.1
Short term	13	0.83 (0.71–0.97) *	66.8
Jadad score			
High quality	9	0.88 (0.73–1.05)	68.7
Low quality	4	0.71 (0.57–0.89) *	26.7
Cochrane ROB			
High quality	5	0.93 (0.74–1.16)	43.6
Low quality	8	0.78 (0.64–0.97) *	72.9
Regimen			
Daily	15	0.83 (0.73–0.95) *	69.1
Jadad score			
High quality	10	0.89 (0.78–1.02)	67.0
Low quality	5	0.69 (0.58–0.82) *	0.0
Cochrane ROB			
High quality	4	0.87 (0.66–1.15)	51.0
Low quality	11	0.81 (0.69–0.96) *	74.9
Weekly	3	1.10 (0.95–1.26)	25.0
Monthly	10	1.00 (0.98–1.02)	0.0
Dose			
High does (>2000 IU)	8	0.95 (0.88–1.02)	57.3
Low dose (≤2000 IU)	20	0.92 (0.85–1.00) * (0.997)	59.5
Type of Disease			
URI	24	0.97 (0.91–1.03)	53.9
LRI	7	1.00 (0.91–1.11)	0.0
Number of study participants			
>1000	6	1.00 (0.98–1.04)	0.0
≤1000	24	0.92 (0.85–0.99) *	68.7
Region			
America (Canada, U.S.)	6	0.93 (0.84–1.03)	0.0
Europe (Finland, Sweden, UK)	5	0.97 (0.86–1.09)	36.9
Asia (Afghanistan, China, India, Israel, Japan, Mongolia, Vietnam)	11	0.85 (0.69–1.05)	74.3
Oceania (Australia, New Zealand)	4	1.00 (0.98–1.03	0.0
Type of prevention			
Primary prevention	26	0.94 (0.89–0.99) *	58.5
Secondary prevention	4	1.05 (0.92–1.21)	59.2
Mean age			
Children	12	0.87 (0.75–1.02)	70.9
Adults	18	0.99 (0.95–1.04)	41.1
Funding source			
Pharmaceutical company	8	0.99 (0.93–1.04)	0.0
Not pharmaceutical company	22	0.94 (0.87–1.00)	69.9
Use of placebo	29	0.98 (0.94–1.02)	44.2
Quality			
Jadad score			
High quality (≥5)	18	1.00 (0.97–1.02)	0.0
Low quality (<5)	12	0.85 (0.69–1.04)	80.6
Cochrane ROB			
High quality (>5)	14	1.00 (0.96–1.03)	10.9
Low quality (≤5)	16	0.90 (0.81–1.01)	73.5

ARI, acute respiratory infection; RCT, randomized controlled trials; RR, relative risk; ROB, risk of bias; URI, upper respiratory tract infection; LRI, lower respiratory tract infection; CI, confidence interval. * Indicates a statistically significant association.

## Data Availability

The authors used published data from the individual studies and declare that the data supporting the findings of this study are available within the article.
